# CHAC1 as a novel biomarker for distinguishing alopecia from other dermatological diseases and determining its severity

**DOI:** 10.1049/syb2.12048

**Published:** 2022-08-18

**Authors:** Hassan Karami, Samira Nomiri, Mohammad Ghasemigol, Niloufar Mehrvarzian, Afshin Derakhshani, Mohammad Fereidouni, Masoud Mirimoghaddam, Hossein Safarpour

**Affiliations:** ^1^ Student Research Committee Faculty of Medicine Birjand University of Medical Sciences Birjand Iran; ^2^ Department of Biochemistry Faculty of Medicine Birjand University of Medical Sciences Birjand Iran; ^3^ Department of Computer Engineering University of Birjand Birjand Iran; ^4^ Department of Pharmaceutical Nanotechnology Faculty of Pharmacy Mashhad University of Medical Sciences Mashhad Iran; ^5^ McCaig Institute, Hotchkiss Brain Institute Snyder Institute for Chronic Diseases University of Calgary Calgary, Alberta Canada; ^6^ Department of Biochemistry and Molecular Biology University of Calgary Calgary Alberta Canada; ^7^ Cellular and Molecular Research Center Birjand University of Medical Sciences Birjand Iran; ^8^ Department of Dentistry University of Alberta Edmonton Alberta Canada

**Keywords:** alopecia areata, drug repositioning, machine learning, molecular pathogenicity, transcriptome analysis, WGCNA

## Abstract

Alopecia Areata (AA) is characterised by an autoimmune response to hair follicles (HFs) and its exact pathobiology remains unclear. The current study aims to look into the molecular changes in the skin of AA patients as well as the potential underlying molecular mechanisms of AA in order to identify potential candidates for early detection and treatment of AA. We applied Weighted Gene Co‐expression Network Analysis (WGCNA) to identify key modules, hub genes, and mRNA–miRNA regulatory networks associated with AA. Furthermore, Chi2 as a machine‐learning algorithm was used to compute the gene importance in AA. Finally, drug‐target construction revealed the potential of repositioning drugs for the treatment of AA. Our analysis using four AA data sets established a network strongly correlated to AA pathogenicity based on *GZMA*, *OXCT2*, *HOXC13*, *KRT40*, *COMP*, *CHAC1*, and *KRT83* hub genes. Interestingly, machine learning introduced these genes as important in AA pathogenicity. Besides that, using another ten data sets, we showed that *CHAC1* could clearly distinguish AA from similar clinical phenotypes, such as scarring alopecia due to psoriasis. Also, two FDA‐approved drug candidates and 30 experimentally validated miRNAs were identified that affected the co‐expression network. Using transcriptome analysis, suggested *CHAC1* as a potential diagnostic predictor to diagnose AA.

## 1 INTRODUCTION

Alopecia Areata (AA) is a common, polygenic, non‐scarring dermatologic autoimmune disease that occurs after immune infiltrates around the hair follicles (HFs) in the actively growing anagen phase and results in hair loss of varying severity that may persist for life [1]. It is the most prevalent autoimmune disease that affects both males and females in children and adults and hair of all colours [2]. It presents with round patches of hair loss on the scalp and can progress to Alopecia Totalis (AT) and Alopecia Universalis (AU) [3]. AA affects 2% of the population worldwide, and its incidence in adults is lower than in children, and it is rising over time and varies significantly by area [4]. Though AA is not life‐threatening, it can bring considerable psychological stress to patients and seriously impact their quality of life [5]. The pathobiology of AA is incompletely understood. Lesional biopsies of AA patients have shown a decrease in the anagen‐to‐telogen ratio [6], rise of CD8+ T cells, inflammatory markers (*IL‐2*, *IL2RA*, *JAK3*, and *IL‐15*), *T* helper type (Th) 1 pathway cytokines (*IL‐12*/*IL‐23p40*, *CXCL10*, *CXCL9*, and *IFN‐gþ*), and Th2 pathway cytokines (*IL‐13*, *IL‐32*, *CCL17*, and *CCL18*), and keratins downregulation (*KRT35*, *KRT75*, and *KRT86*) [7]. Upregulation of the phosphosignal transducer and transcription activator (STAT)1/pSTAT3 was also detected in HFs of AA patients but not in uninfluenced controls [8]. Furthermore, environmental insults, such as viral infections, trauma, and genetic predisposition, can be effective [1, 9]. To date, there are no FDA‐approved drugs for AA. Multiple empiric options, including observation, intralesional steroids, topical immunotherapy, or broad immunosuppressive, are used for AA treatment [1]. As the molecular mechanism underlying these diseases is not entirely defined [1, 10], more research is needed to uncover the molecular mechanism and identify new promising therapeutic targets for AA. Recently, some studies investigated the roles of some specific molecules [11, 12] and several genes [13, 14] in the pathogenesis of AA. Because of the large amounts of data produced by RNA‐Seq and micro‐array technologies, new approaches are required to effectively extract meaningful associations from highly multivariate data sets [5, 6]. Numerous classification and evaluation metrics for identifying differentially expressed genes (DEGs) in microarray data have been investigated [15]. There are several methods for assessing DEGs in different diseases like AA. Several machine learning algorithms, such as support vector machines, random forests, and Chi2 classifiers are used for the prediction of biomarkers for specific diagnosis of the disease [16, 17]. Weighted gene co‐expression network analysis (WGCNA) is considered a bioinformatic tool for exploring intrinsic transcriptome organisation [18, 19]. WGCNA can be used to find modules of highly correlated genes for summarising such clusters using the module Eigengene (ME) or an intramodular hub gene to relate modules to each other and external sample characteristics. Finally, candidate biomarkers or therapeutic targets can be identified using correlation networks [20, 21]. This method has been successfully applied before in diverse biological contexts to identify regulatory genes and networks [22]. In the current in silico work, we applied two different computational methods, machine learning and systems biology approaches, for analysing a microarray data set from the Gene Expression Omnibus (GEO) database from AA patients and healthy controls to investigate the molecular level changes in the skin of AA patients and the potential underlying molecular mechanisms of AA to determine the putative candidates for early detection and treatment of AA.

## 2 MATERIALS AND METHODS

### 2.1 Data set identification and preprocessing

We used four previously published microarray gene expression data sets from the GEO database (https://www.ncbi.nlm.nih.gov/geo/) for skin samples from 19 AA patients and 14 controls (Table 1). These data sets were based on the GPL570 [HG‐U133_Plus_2] platform. First, for each of the data sets, the raw data were log2 transformed, quantile‐normalised, and probe IDs were converted to gene symbols. When multiple probes were mapped to the same gene, median values were used to represent the expression of that gene. Consequently, the batch correction was performed on the four data sets based on the expression of common genes using ‘combat’ and ‘sva’ (SVA *R* package) functions. Finally, gene symbols were filtered across all samples through their variance. Only genes with variances ranked in the top 4000 were selected for subsequent analyses.

**TABLE 1:** Data set information

ID in GEO	Platform ID	Sample type	Samples^a^	Reference
GSE45512	GPL570	Scalp skin biopsies	10	(23)
GSE80342			12	(24)
GSE58573			5	(23)
GSE74761			6	(25)

^a^ Indicates the number of samples included in our study.

### 2.2 Identification of differentially expressed genes

The R/Bioconductor package ‘Limma’ version 3.28.14 was used to screen DEGs between AA and normal groups from 4 data sets [26]. The raw data were corrected, and quantile‐normalisation was performed using the using *normalize Quantiles* function of the limma package. Genes with Adjust *p*‐value < 0.01 and |log2FC| ≥ 2 were considered as differentially expressed. The biological processes (BP) and Kyoto Encyclopedia of Genes and Genomes (KEGG) pathway enrichment analysis were also conducted, and corresponding results were structured and visualised by the Clupedia plugin of Cytoscape software 3.0. Cluepedia integrates and summarises enriched terms into major biological networks via KEGG/BioCarta pathways and creates an applicable, organised, and functional annotation term network.

### 2.3 Construct co‐expression modules of AA

The *R*‐based package of WGCNA version 1.63 was used for the construction of the gene co‐expression network of patients and control groups [27]. Shortly, according to the Pearson test, the matrix of the gene expression profile was converted to the matrix of pairwise gene similarity, accompanied by a translation to the adjacency matrix. According to the scale‐free gene co‐expression of the topological algorithm already defined, the adjacency matrix met the scale‐free topology criterion when the *β* value is considered to be 5. After that, the Topological Overlap Matrix (TOM) and dissimilarity TOM (dissTOM) were generated using similarity and dissimilarity modules in TOM. Finally, a minimum module size of 30 genes and a cut height of 0.32 were considered for the creation of the clusters of highly interconnected genes.

### 2.4 Construct module‐trait relationships of AA

In order to recognise modules that were significantly related to the evaluated clinical trait, expression profiles of each module were summarised via its ME as the eigenvector correlated to the first principal component of the expression matrix. The relationship between MEs and the clinical feature was assessed by the Pearson test, and if *p* < 0.05, then the module and clinical trait were regarded as a statistical correlation. The gene significance (G.S) values were used for measuring individual genes’ associations with the AA. Also, module membership (M.M) was defined as the correlation of the ME and the gene expression profile for each module. If the G.S and M.M were positively correlated, the most significant (central) elements in the modules were closely associated with the trait [28]. So, they can be used to construct the network and identify the hub genes.

### 2.5 Module preservation analysis

To confirm the reliability of the recognised module with a significant correlation to AA, we performed the module preservation analysis using GSE68801 data sets. Preservation analysis is based on estimating gene correlation with the ME, differences between what is observed and what is obtained by random permutation. One can check whether the values found in the reference network are correlated for the same genes within the other network. In this regard, the first 4000 genes with the highest coefficient of variation were used as an input to assess the level of module preservation in each data set. The degree of module preservation was measured through *Z*_summary_ statistics, in which *Z*_summary_ < 2 shows no preservation, 2 < Z_summary_ < 10 indicates weak–moderate preservation, and *Z*_summary_ > 10 suggested strong evidence for preservation.

### 2.6 Feature selection by Chi2

Chi2 is another popular feature selection method that can be used to eliminate some irrelevant attributes. The $\chi^{2}$ test is a statistical test applied to determine the dependency of two events. The following formula is used to compute $\chi^{2}$:

(1) $$\chi_{c}^{2}=\sum_{i=1}^{2}\sum_{j=1}^{k}\frac{{\left(A_{ij}-E_{ij}\right)}^{2}}{E_{ij}}$$

(2) c=(n−1)(m−1)

where c is the degree of freedom, n is the number of samples, m is the number of attributes, A stands for actual and E stands for expected value. To apply the $\chi^{2}$ test for feature selection, we can calculate the $\chi^{2}$ value between each feature and the target class. Then, we can select the desired number of features with the best $\chi^{2}$ scores. In other words, if a feature is independent of the target, it is not informative in the classifying process.

In this paper, we face a huge data set with many genes as the features. Hence, we apply the $\chi^{2}$ feature selection algorithm [17] to select the most important genes. The algorithm begins with a high significance level (sigLevel) for all numeric attributes for discretisation. Each attribute is sorted according to its values. Then, we calculate the $\chi^{2}$ value for every pair of adjacent intervals with the following equation (at the beginning, each pattern is put into its own interval that contains only one value of an attribute).

(3) $$\chi^{2}=\sum_{i=1}^{2}\sum_{j=1}^{k}\frac{{\left(A_{ij}-E_{ij}\right)}^{2}}{E_{ij}}$$

(4) Eij=Ri∗C/jN

(5) $$R_{i}=\sum_{i=1}^{k}A_{ij}$$

(6) $$C_{i}=\sum_{i=1}^{2}A_{ij}$$

(7) $$N=\sum_{i=1}^{2}R_{i}$$

where k is the number of classes, $A_{ij}$ is the number of patterns in the *i*th interval and *j*th class, $\;E_{ij}$ is the expected frequency of $A_{ij}$, $R_{i}$ is the number of patterns in the *i*th interval, and $C_{j}$ is the number of patterns in the *j*th class.

Afterwards, we merge the pair of adjacent intervals with the lowest $\chi^{2}$ value. Merging continues until all pairs of intervals have $\chi^{2}$ values exceeding the parameter determined by sigLevel. The above process is repeated with a decreased sigLevel until an inconsistency rate δ is exceeded in the discretised data. Algorithm 1 shows the $\chi^{2}$ discretisation and feature selection process with more details.

Algorithm 1: **$\chi^{2}$ Discretisation and Feature selection algorithm**

1

**Phase 1**: 

Initialise significance level to a high value (e.g. sigLevel = 0.5)

While the inconsistency rate in data is less than threshold δ.

 For all continuous attributes

  Sort training data based on attribute

  Create an interval for each instance of the training data, and initialise the intervals.

  While intervals can be merged (some $\chi^{2}$ value is below the threshold).

   Calculate the $\chi^{2}$ value for each pair of adjacent intervals using Equation (3).

   Select the pair with the lowest $\chi^{2}$ value and merge this pair into one interval.

 Decrease the significance level.

**Phase 2:**

Starting with the lowest significance level of phase 1, associate each attribute with the level. For all mergeable attributes, until no attributes can be merged

 Sort training data based on the attribute

 Create an interval for each instance of the training data, and initialise the intervals.

 While intervals can be merged (some $\chi^{2}$ value is below the threshold).

  Calculate the $\chi^{2}$ value for each pair of adjacent intervals using Equation (3).

  Select the pair with the lowest $\chi^{2}$ value and merge this pair into one interval.

 If inconsistency rate in training data does not exceed predefined threshold δ,

  then decrease significance level for this attribute

  else mark the attribute as non‐mergeable.

### 2.7 Functional enrichment analysis of significant module

Functional enrichment analysis of qualified modules was performed using Cluepedia software. Enriched ontological terms and pathways with the threshold of Benjamin‐adjusted *p*‐value < 0.05 were selected.

### 2.8 Hub‐gene detection and co‐expression network reconstruction

Genes with both G.S and M.M ≥ 0.85 were chosen as hub genes if differentially expressed compared to control samples. A Venn diagram was generated using the ‘Venny’ *v* 2.1 software, available freely at (http://bioinfogp.cnb.csic.es/tools/venny/). Functional networks were constructed by GeneMANIA (https://genemania.org/) and visualised using Cytoscape *v* 3.0 software [29].

### 2.9 Evaluation of selected hub genes behaviour in other dermatological diseases

In order to evaluate the expression behaviour of selected hub genes in other skin lesions clinically similar to AA, including psoriatic alopecia and androgenic alopecia (AGA), we performed DEGs analysis on related data sets, including GSE78097, GSE75890, GSE14905, GSE78097, GSE79704, GSE109248, GSE52471, and GSE90594, in the same way mentioned in 2.2 [28].

### 2.10 Evaluation of selected hub‐gene behaviour in other types of AA

To further evaluate the role of selected hub genes in alopecia pathogenicity, we used GSE68801 as another AA data set for WGCNA network reconstruction. This data set contained the most extensive AA samples with specific disease staging to patch‐type AA (AAP), AT, and AU. This analysis aimed to find out the expression behaviour of similar hub genes between the two data sets.

### 2.11 Identification of candidate regulatory miRNAs and drugs

The miRNA regulatory network was built for recognised hub genes using the miRTarBase database (http://mirtarbase.cuhk.edu.cn/php/index.php). Also, the well‐known Drug‐Gene Interaction Database (DGIDB) (http://www.dgidb.org/) was used to connect functional and FDA‐approved drug‐able hub genes [30].

## 3 RESULTS

### 3.1 Preprocessing and identification of DEGs

**FIGURE 1:**
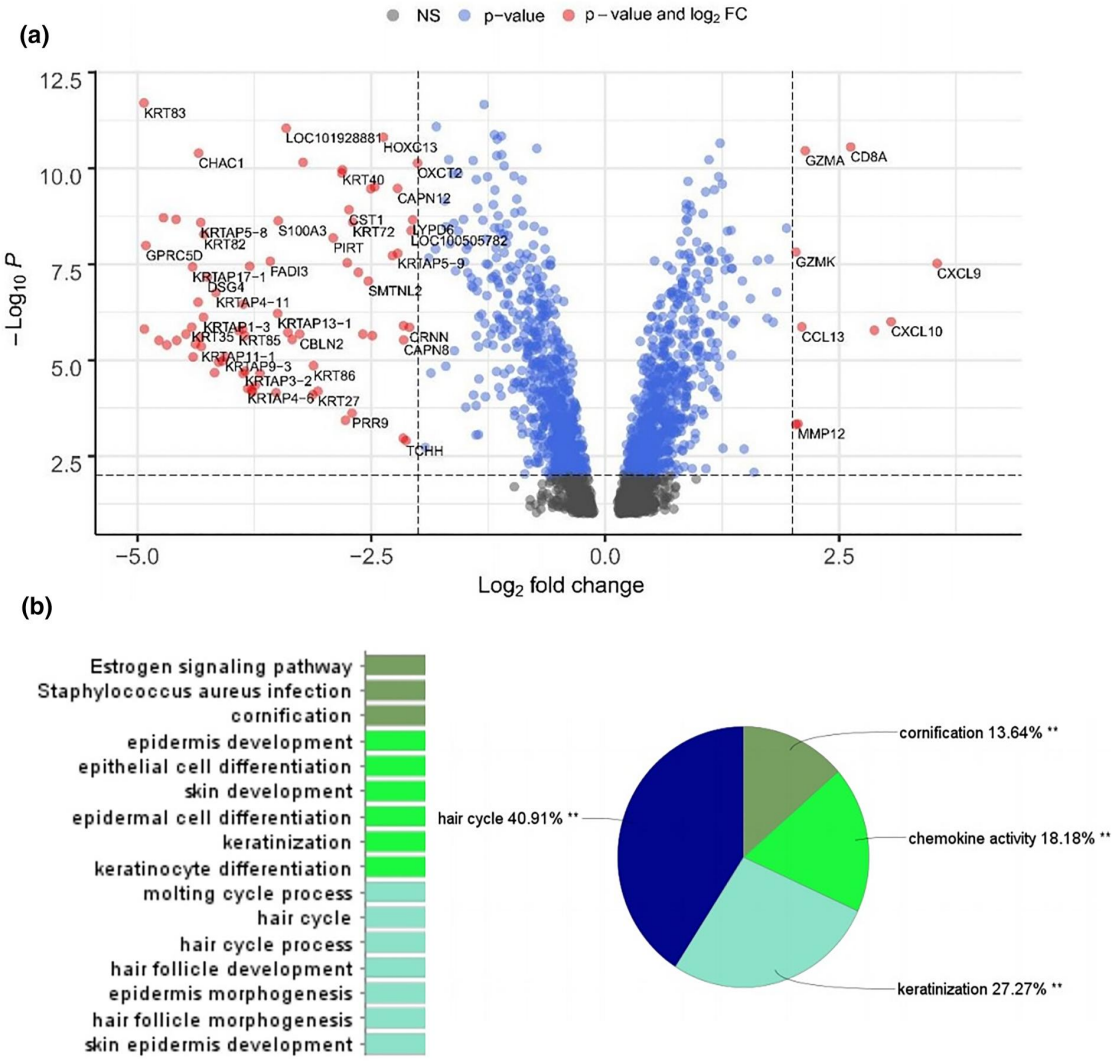
Characterisation of differentially expressed genes (DEGs) in Alopecia Areata (AA). DEG volcano plot (a). This graph represents the genes that are upregulated and downregulated in AA samples. The fold change of genes is displayed by the horizontal axis. The −log10*P*‐value, as determined by the student's *t*‐test, is reflected on the vertical axis. (b) the functional group pie chart, which includes specific annotation terms identified within the DEGs. As previously stated, twenty‐two BP terms and pathways are related to DEGs, which are summarised in four major biological networks, including the hair cycle (40.91 percent), keratinisation (27.27 percent), chemokine activity (18.18 percent), and cornification (13.64 percent)

Preprocessing of data, including quantile normalisation, was performed to reduce the effects of technical noises. A total of 82 genes were identified as DEGs with the threshold of Adjust *p*‐value < 0.01, and |log2FC| ≥ 2, including nine upregulated and 73 downregulated genes (Figure 1a). These 85 DEGs were then nominated for downstream analysis. Hair cycle, keratinisation, and cornification are the most important biological functions and pathways of the DEGs (Figure 1b).

### 3.2 Identification of associated modules with AA pathogenicity

**FIGURE 2:**
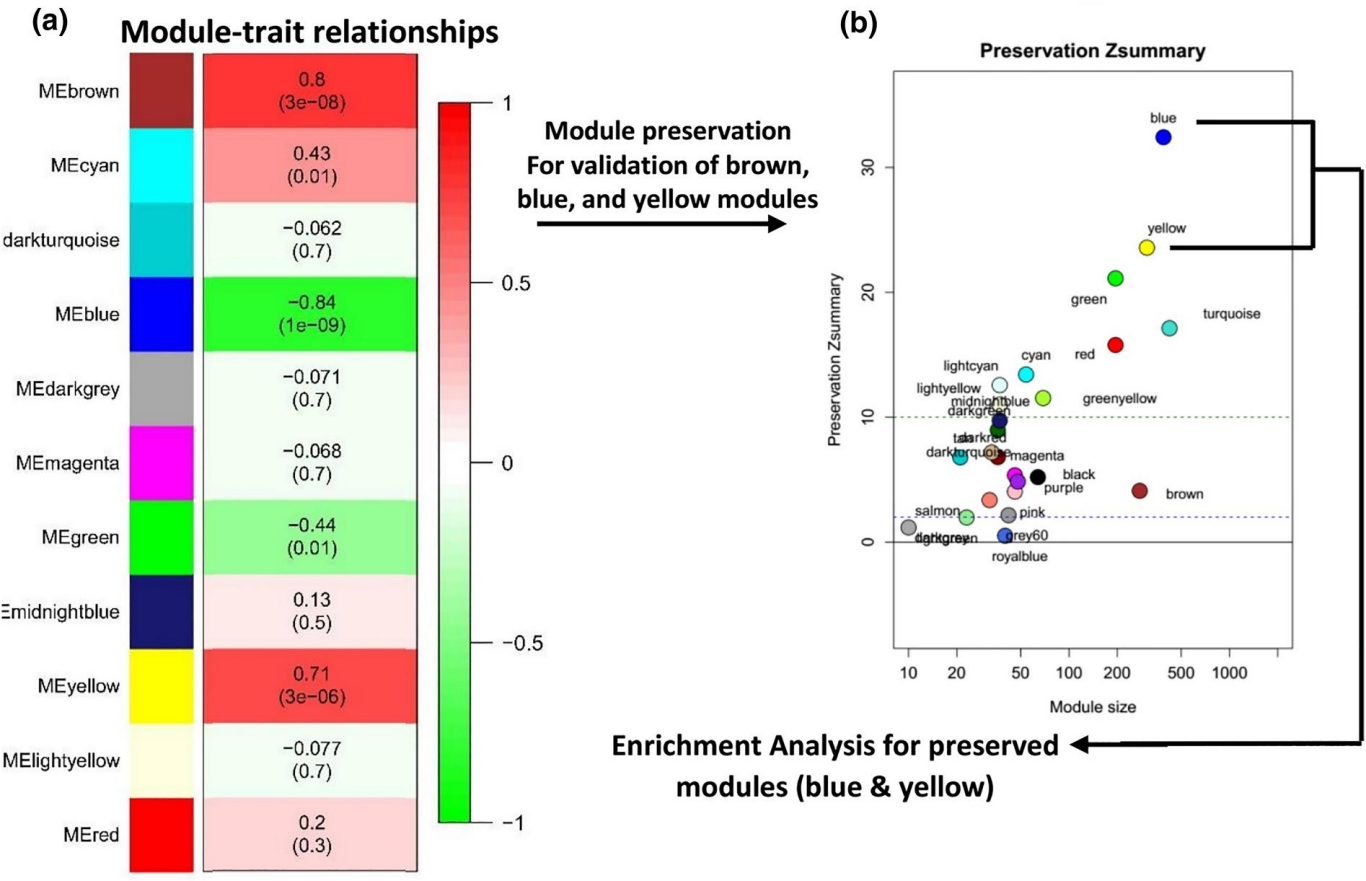
(a) Module‐trait relationship and enrichment analysis of an interested module, (a): Each row corresponds to a module Eigengene (ME), and the column corresponds to Alopecia Areata (AA) status. Numbers indicate the corresponding correlation and *p*‐value in each cell; (b): Module preservation analysis. The GSE68801 data set was used to analyse the preservation attribute. *Z*_summary_2 demonstrates no preservation, 2*Z*_summary_10 indicates weak‐moderate preservation, and *Z*_summary_ > 10 exhibits higher preservation. The blue and yellow modules with a significant correlation and a *Z*_summary_ > 10 were chosen for further analysis

A total of 4000 genes were included in WGCNA based on a variance of expression values. No outlier was found by sample clustering in 33 samples (Figure S1). Subsequently, *β* = 5 was identified as a soft‐threshold power for the construction of a weighted co‐expression network (Figure S2). As a result, the hierarchical clustering dendrogram identified 11 modules illustrated in the branches of the dendrogram with different colours (Figure S3). To consider the association of the modules with the presence of AA in samples and module–module correlation, eigengenes were calculated for each module. As was indicated, blue, brown, and yellow modules were highly correlated with AA disease (Figure 2a and Table 2). According to the module preservation analysis result, with a *Z*_summary_ of 4, the brown module could not meet the criteria and failed to qualify for downstream analysis. On the other hand, the blue and yellow modules with *Z*_summary_ equal to 30 and 23, respectively, were selected for subsequent enrichment and hub‐gene selection (Figure 2b).

**TABLE 2:** Module colour characteristics. The number of genes on each module was varied from 31 (dark grey) to 1414 (blue). According to module preservation results, the blue and yellow modules with maximum *Z*_summary_ were selected for subsequent enrichment and hub‐gene selection

Module colours	# Genes	Correlation	*p*‐value	*Z*_summary_	Qualified
Blue	1414	−0.84	1.00E‐09	30	✓
Brown	486	0.8	3.00E‐08	4.1	×
Cyan	496	0.43	0.01	13	×
Dark grey	31	−0.071	0.7	1.2	×
Dark turquoise	33	−0.062	0.7	6.8	×
Green	448	−0.44	0.01	22	×
Light yellow	45	−0.077	0.7	11	×
Magenta	96	−0.068	0.7	5.4	×
Midnight blue	195	0.13	0.5	16	×
Red	297	0.2	0.3	16	×
Yellow	459	0.71	3.00E‐06	24	✓

### 3.3 GO and KEGG analysis of qualified modules

**FIGURE 3:**
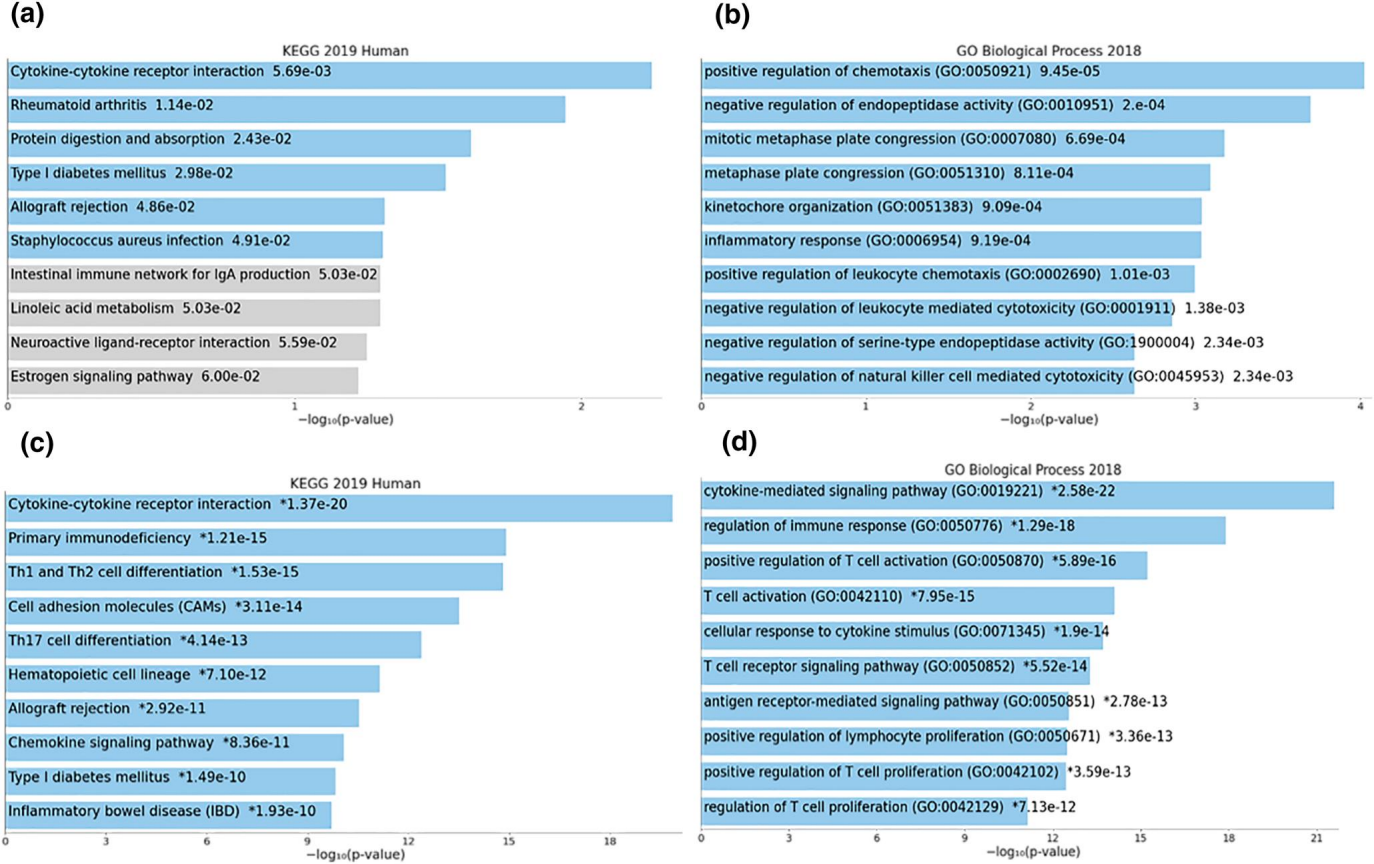
KEGG and GO functional enrichment analysis of qualified modules. In the blue module: (a) KEGG and (b) BP analysis results. In yellow module: (c) KEGG and (d) BP analysis results

The critical biological process and KEGG pathways related to the selected modules were visualised using the EnrichR in Figure 3. The most significant pathway associated with the blue and yellow modules was cytokine–cytokine receptor interaction. On the other hand, positive regulation of chemotaxis, negative regulation of endopeptidase activity, and mitotic metaphase plate congression were the most important biological functions of the blue module genes. The yellow module genes were mostly in the biological process, including the cytokine‐mediated signalling pathway, regulation of immune response, and positive regulation of T cell activation.

### 3.4 Chi2 as a machine learning algorithm

**FIGURE 4:**
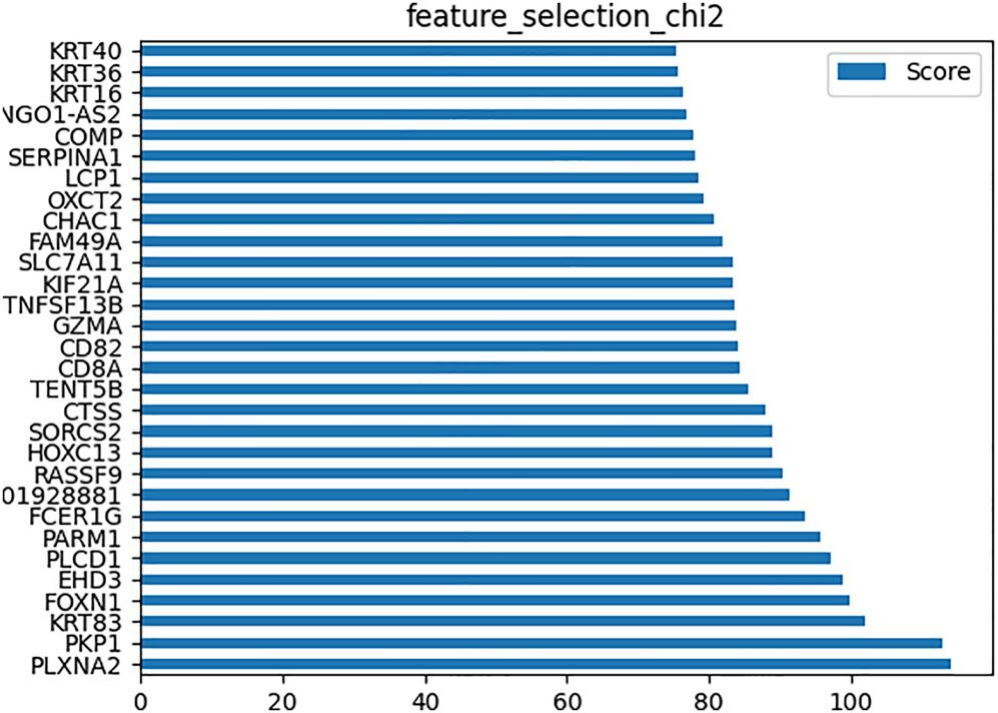
The gene importance computation using χ^2 Discretisation and feature selection algorithm

Figure 4 shows the result of extracting the 30 essential genes from the first 4000 genes of the four mentioned data sets. These genes were then compared to those genes that were selected from WGCNA for hub‐gene selection.

### 3.5 Hub‐gene detection and co‐expression network reconstruction

**FIGURE 5:**
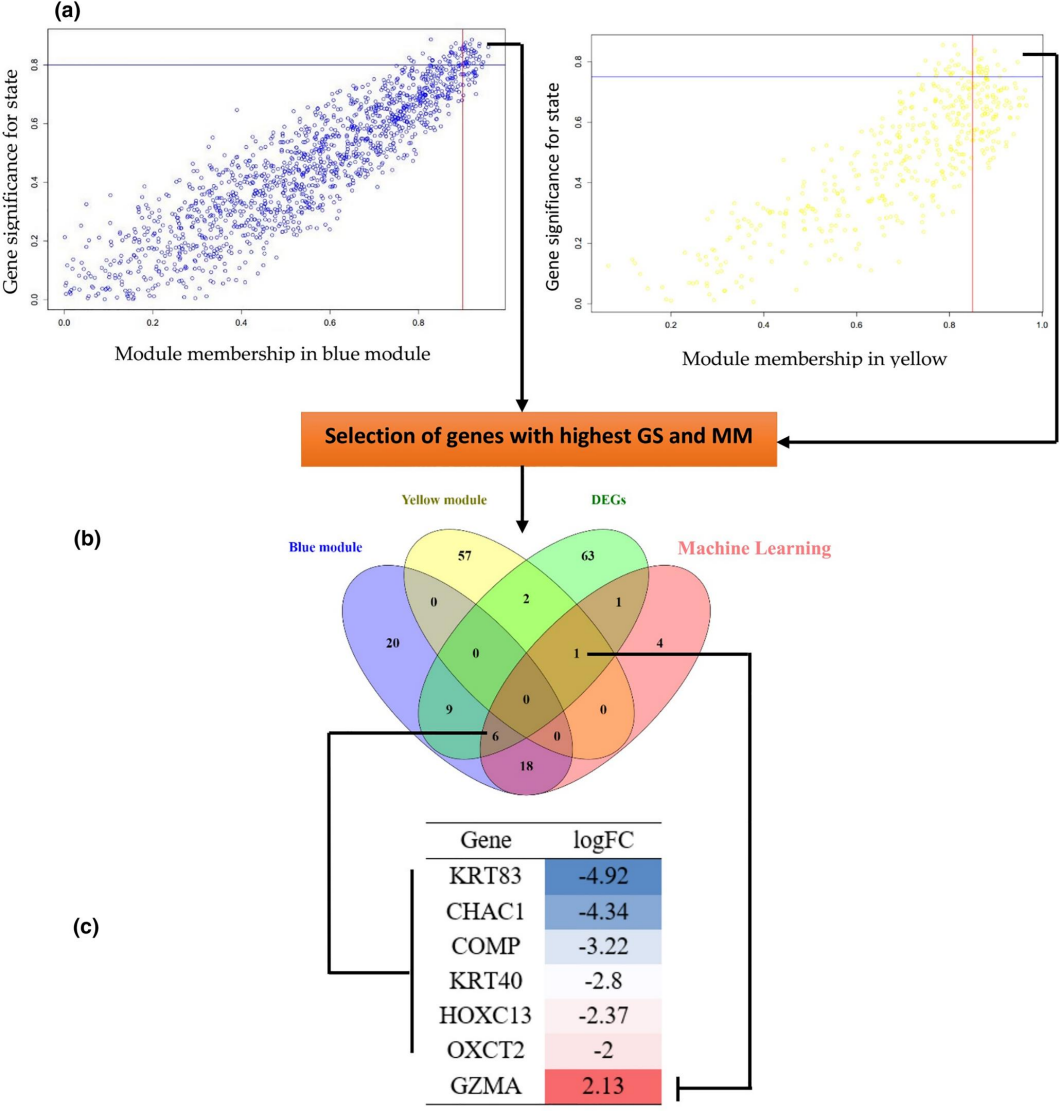
(a): Each module's GS and MM characteristics (a): Modules strongly associated with Alopecia Areata (AA) status (control vs. patient) Each point represents an individual gene within each module, which is plotted on the *y*‐axis by GS and the *x*‐axis by MM; (b): A comparison of blue and yellow module hub genes and differentially expressed genes (DEGs). To construct the co‐expression network, similar genes between lists were selected. Following the selection of these similar genes, the GeneMANIA database was used for co‐expression network construction; (c): Log2FC of chosen hub genes

As previously explained in the M&M section, G.S is defined as the absolute value describing the relationship between the gene and the clinical trait, while the M.M describes the correlation between the eigengene and the gene expression profile. Thus, gene candidates were searched among the top‐scoring gene according to M.M and G.S parameters. The correlation between features (M.M and G.S) of the selected modules (Figure 5a) led to the detection of hub genes of interest that were highly associated with AA pathogenesis. The genes with maximum M.M and G.S scores in each module were then compared to the DEGs list, and similar genes were considered as final hub genes (Figure 5b). These final hub genes included *GZMA*, *OXCT2*, *HOXC13*, *KRT40*, *COMP*, *CHAC1*, and *KRT83* (Figure 5c). The co‐expression network of the blue and yellow module hub genes was reconstructed using GeneMANIA and Cytoscape software.

### 3.6 Evaluation of selected hub genes behaviour in other dermatological diseases

**FIGURE 6:**
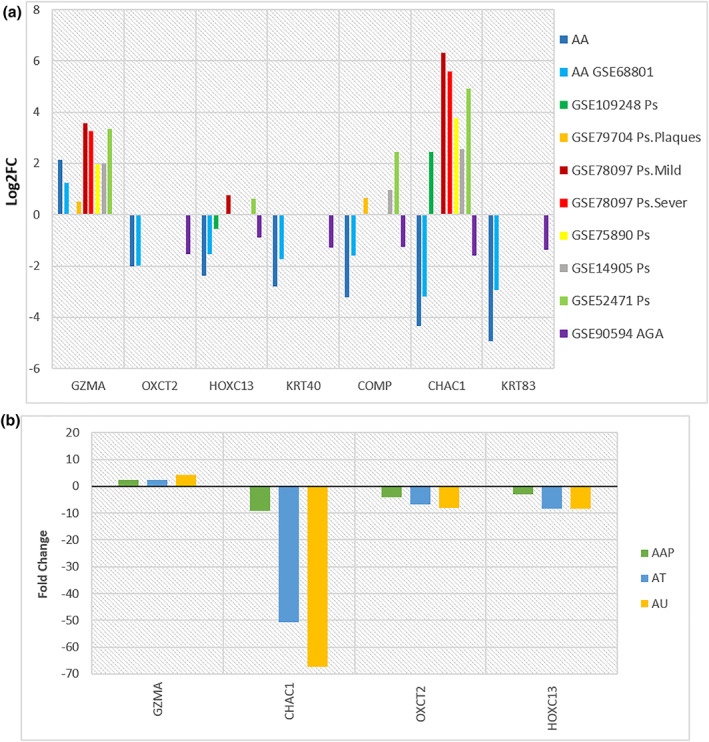
(a): Expression levels of selected hub genes in common hair loss diseases such as Alopecia Areata (AA), androgenic alopecia (AGA), and psoriasis; (b): Transcriptional changes of common hub genes with GSE68801 in another type of alopecia

The expression patterns of all seven selected hub genes from AA data sets were compared to those of psoriasis and AGA in 7 related data sets. As indicated in Figure 6a, all selected hub genes present a similar expression pattern among AA, psoriasis, and AGA data sets, except four genes, including *CHAC1*, *COMP*, and *HOXC13*. Among them, *CHAC1* has a significant difference between AA and psoriasis data sets. These findings indicated a potential role for the *CHAC1* gene in distinguishing AA from psoriatic alopecia.

### 3.7 Evaluation of selected hub genes behaviour in other types of AA

The WGCNA analysis of GSE68801 identified two new modules that were significantly correlated to the AAP trait (data not shown). Functional enrichment of these modules revealed biological pathways shared with blue and yellow modules that were closely related to AA development [1]. For instance, dysregulation of immune regulatory switch‐off mechanisms that limit the course of inflammation and cytokine production to prevent excessive tissue damage is the main physiopathology of autoimmune disorders such as AA [2]. Moreover, immune cell filtration around HFs is prominently made up of T cell subpopulations, which exhibit antagonistic activities in terms of activation or suppression of the exciting inflammation [31]. Notably, several studies support the contribution of *IFN‐γ*‐driven immune response as the primary driver of AA pathogenesis by CD8+ cytotoxic T cell recruitment to impair the maintenance of the immune privilege of the HF [32].

Moreover, network analysis and hub‐gene detection of selected modules from GSE49451 revealed that *GZMA, OXCT2*, *HOXC13,* and *CHAC1* hub genes were shared between two data sets, robustly confirming significant implication in AA development.

To better understand the exact molecular mechanism of these seven common hub genes in AA development, the transcriptional profile of these hub genes in the AAP group was compared to that of AT and AU groups. According to Figure 6b, a comparison of transcriptional changes in lesion skin biopsies revealed that *GZMA* was positive, and *OXCT2, HOXC13,* and *CHAC1* were negatively associated with inflammation status and disease severity, which indicates a mechanistic role of these chemokines in AA development.

### 3.8 MicroRNAs as upstream regulators for common hub genes

**FIGURE 7:**
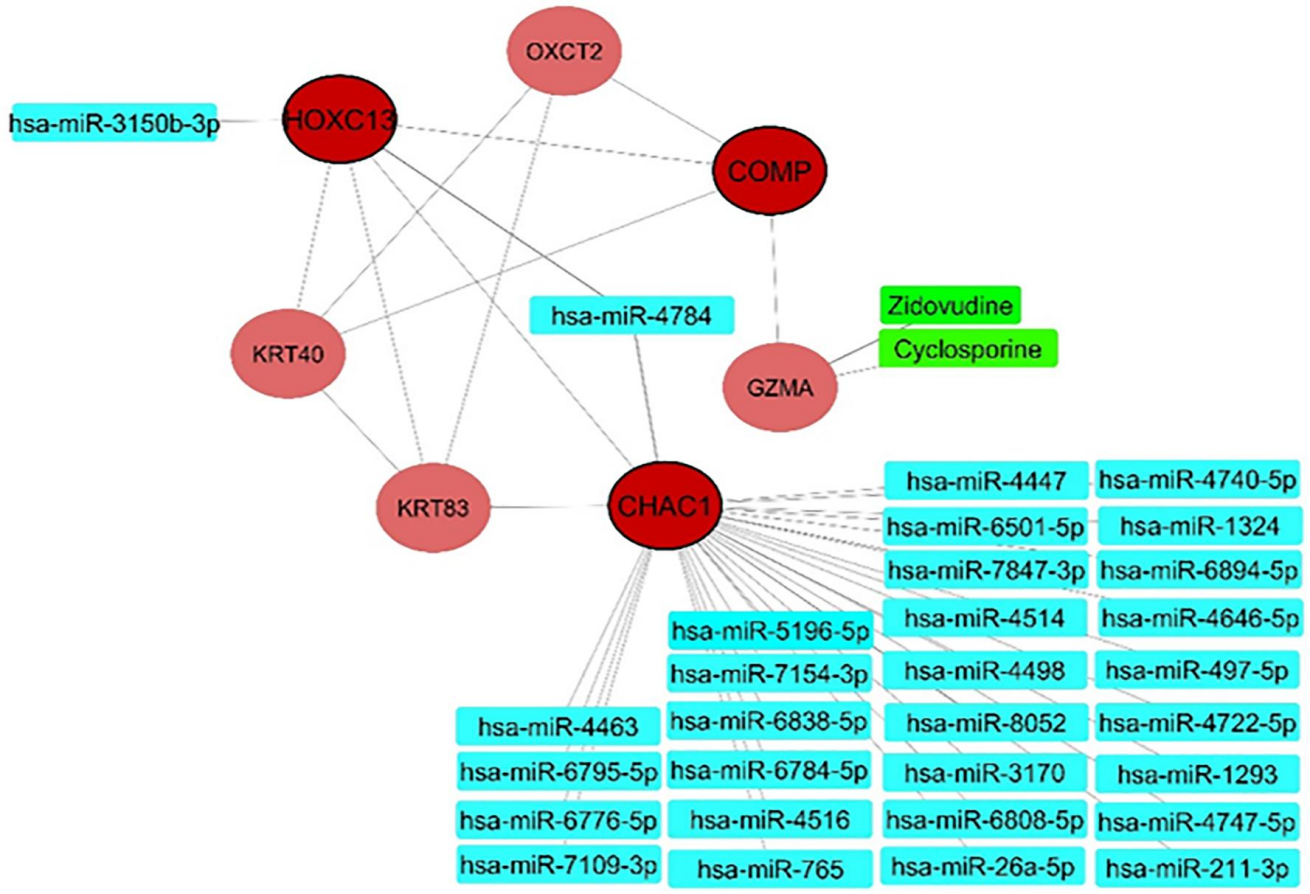
A network of selected hub genes' miRNA–mRNA and drug‐target interactions. For each selected hub gene, experimentally validated miRNAs (turquoise) were obtained from the miRTarBase database, and FDA‐approved drugs (green) were obtained from the DGIDB database

To identify the possible molecular signature of the hub genes, their predicted miRNAs have been analysed via miRWalk database. Figure 7 displays experimentally validated miRNAs of selected hub genes. As a result, the two hub genes regulated by these miRNAs were *HOXC13* and *CHAC1*.

### 3.9 Drug‐target network construction

To analyse the drug development perspective of the blue module, we tested if it harbours known targets of AA drugs. This target in the module included *GZMA* (Cyclosporine and Zidovudine) (Figure 7). The presence of the target in the module of interest suggested that these drugs potentially impact AA and could be considered as possible candidates for further research in this respect.

## 4 DISCUSSION

Alopecia Areata (AA) is an autoimmune skin disease characterised by non‐scarring hair loss resulting from damage to the HF immune privilege by T cells, leading to non‐scarring hair loss and small patches of baldness on the scalp and/or entire body [33]. The lack of knowledge regarding precise pathophysiology, unpredictable disease onset and progression, absence of preventive and effective drugs as well as adverse effects of current treatments highlights the importance of better understanding the molecular basis of the disease and recognising putative treatment targets [33, 34].

Histologically, AA is characterised by an immune infiltrate centred around the hair bulb. Substantial differences in histological appearance have not been described when comparing AAP, AT, and AU samples. However, others have cited that disease duration may impact the amount of peribulbar infiltrate, with more acute cases being reported as having relatively more robust inflammation and chronic cases as having less [1]. On the other hand, despite clinically based diagnosis of AA, distinctive inflammatory and epidermal biomarkers will be helpful for physicians and pathologists to recognise AA among multiple similar scarring and non‐scarring forms of alopecia such as psoriatic alopecia and androgenic alopecia that may resemble clinical features of AA and lead to making appropriate therapeutic choices [35, 36]. Here, we aimed to distinguish this type of autoimmune disease based on the gene expression pattern. Our comprehensive analysis using four AA data sets based on WGCNA and machine learning identified two correlated modules to AA pathogenicity, which leads to the construction of a co‐expression network based on *GZMA*, *OXCT2*, *HOXC13*, *KRT40*, *COMP*, *CHAC1*, and *KRT83* hub genes. It is noteworthy to highlight that the dysregulation of a majority of these hub genes was reported in a recent bioinformatic study [37]. However, only an ordinary DEGs analysis has been performed on two data sets without a cell‐specific genome‐wide method considering gene–gene connectivity patterns and functional gene significance that warrants further studies.

Cation Transport Regulator‐Like Protein 1 (CHAC1) is a recently identified enzyme involved in the γ− glutamyl cycle that takes part in glutathione depletion and the accumulation of reactive oxygen species (ROS), leading to unbalanced cellular redox levels [38, 39]. Furthermore, *CHAC1* was discovered to participate as a component of the unfolded protein response (UPR) pathway, which is a stress‐signalling pathway induced by the presence of misfolded proteins in the endoplasmic reticulum as a result of alternations in the redox state, calcium levels, and other stressful cellular conditions, which in turn, promotes the expression of several pro‐apoptotic proteins, such as *CHAC1,* leading to programmed cell death such as apoptosis or ferroptosis [39, 40, 41]. Interestingly, emerging data suggest that abnormalities of the programmed cell death pathway in response to existing oxidative stress result in the survival of aberrant cells presenting damaged self‐antigens to the immune system and may play an important role in initiating the inflammatory response result in autoimmune diseases, such as AA [42, 43]. Moreover, the downregulation of *CHAC1* in AA‐related microarray data sets has been confirmed by previous in silico studies [37, 44, 45]. These findings strengthen the hypothesis that downregulation of the pro‐apoptotic *CHAC1* in AA lesions may robustly contribute to the activation of the immune system by self‐antigens, breaking immune tolerance, and consequently initiating autoimmunity in AA, triggered by environmental stresses.

Additionally, it has been identified that the overexpression of *CHAC1* is associated with the poor outcomes of various cancers, including uveal melanoma, breast, and ovarian cancer, due to its substantial role in promoting tumour cell proliferation and migration [46, 47]. However, considering the critical involvement of *CHAC1* in glioma apoptotic cell death, the suppression of neuroblastoma cell proliferation and mediating antitumour activity of artesunate, nisin, and temozolomide highlights the opposite regulatory function of *CHAC1* in cell proliferation and migration of different tumour tissues [39, 47, 48]. It is noteworthy that, consistent with previous studies, in contrast to the significant downregulation of *CHAC1* in affected AA skin, we found an increase in *CHAC1* expression in psoriatic epidermis compared with normal skin, which suggests specific pathological mechanisms discriminating AA from other inflammatory hair loss disorders such as psoriasis [37, 49]. However, the possible mechanisms underlying *CHAC1* dysregulation in skin epithelial cells during AA and psoriasis development remained to be elucidated.

Homoeobox Protein Hox‐C13 (*HOXC13*) is a member of the HoxC gene cluster recruited for unique and necessary functions in the development of some ectodermal organs, including hair, nail, and filiform papilla [38, 50]. The fact that both downregulation and upregulation of *HOXC13* affect the regular expression of hard keratins specific to the hair and led to hair loss, as illustrated both by the brittle hair resulting in the alopecia phenotype, displayed by mice lacking the function of *HOXC13,* and by the hairless phenotype of mouse models overexpressing HOXC13, resembling ichthyosis, further highlights the vital regulatory role of *HOXC13* expression levels over various keratin genes [51, 52, 53, 54, 55]. Consistent with our results, *HOXC13* downregulation during AA development has been recently identified using DEG analysis [37, 45].

As depicted in Figure 6, further network‐based drug repositioning is constructed to discover new and potent drugs for AA treatment. However, some of these drug predictions have been shown to be beneficial for AA therapy. For instance, oral and topical Cyclosporine therapy has been used for severe cases, and combination therapy with these drugs has been suggested to be a useful treatment for severe AA [56, 57, 58]. The immunomodulatory properties of statins highlight the efficacy of these drugs against various inflammatory dermatological conditions such as AA, acne, and vitiligo, as reported by some studies [59, 60]. However, the predictable effects of immune‐stimulated agents, including antiretroviral drugs on generalised hair loss and AA development, have been described before following treatment with Zidovudine [61].

Although our *in‐silico* findings may lead to a deeper understanding of AA pathogenicity, we have not verified the biological functions of the selected hub genes in AA. Nor have we determined how suggested miRNAs and approved drugs target the hub genes related to AA. Therefore, more in vitro and in vivo experiments are still needed, and we will continue to focus on this issue in our further studies.

## 5 CONCLUSION

Considering the lack of knowledge regarding precise pathophysiology, unpredictable disease onset and progression, absence of preventive and effective drugs as well as adverse effects of current treatments, we aim to explore the molecular mechanism of AA by incorporating the merits of two different computational methods, machine learning and systems biology approaches, using gene expression microarray data sets. To summarise, our results showed significantly correlated genes, which can be used as candidate genes in AA pathogenicity for further evaluations. Among them, the *CHAC1* gene has the potential to distinguish Psoriatic alopecia from AA and can be used for the determination of alopecia severity. Also, drug‐target network analysis confirmed that 2 FDA‐approved drugs are potential candidates for the treatment of AA patients. Our findings also indicated that the seven experimentally validated miRNAs controlled the co‐expression network through 2 hub genes. Further studies on blood and tissue verification of these hub genes and relative pathways are still needed.

## CONFLICT OF INTEREST

The authors declare that there is no conflict of interest.

## Supporting information

Supplementary Material S1

## Data Availability

The data that support the findings of this study are openly available in GEO database at https://www.ncbi.nlm.nih.gov/geo, reference number [23, 24, 25].
