# Supplementary material for: CHAC1 as a novel biomarker for distinguishing alopecia from other dermatological diseases and determining its severity
Source: IET Syst Biol. 2022 Aug 18;16(5):173–85. doi: 10.1049/syb2.12048 (PMC9469792; doi:10.1049/syb2.12048)

**Supplementary Figs**

**Figure S1:** Clustering of samples to find outliers. The color is relative to the stage of AA pathology (Red=AA samples and white=normal samples);

**Figure S2:** Soft-thresholding powers selection. For downstream analysis, β was set as 5;

**Figure S3:** Cluster dendrogram and module assignment from WGCNA. The divisions belong to closely interlinked gene groups. The modules are depicted in the horizontal bar by colors.

**Fig. S1:**


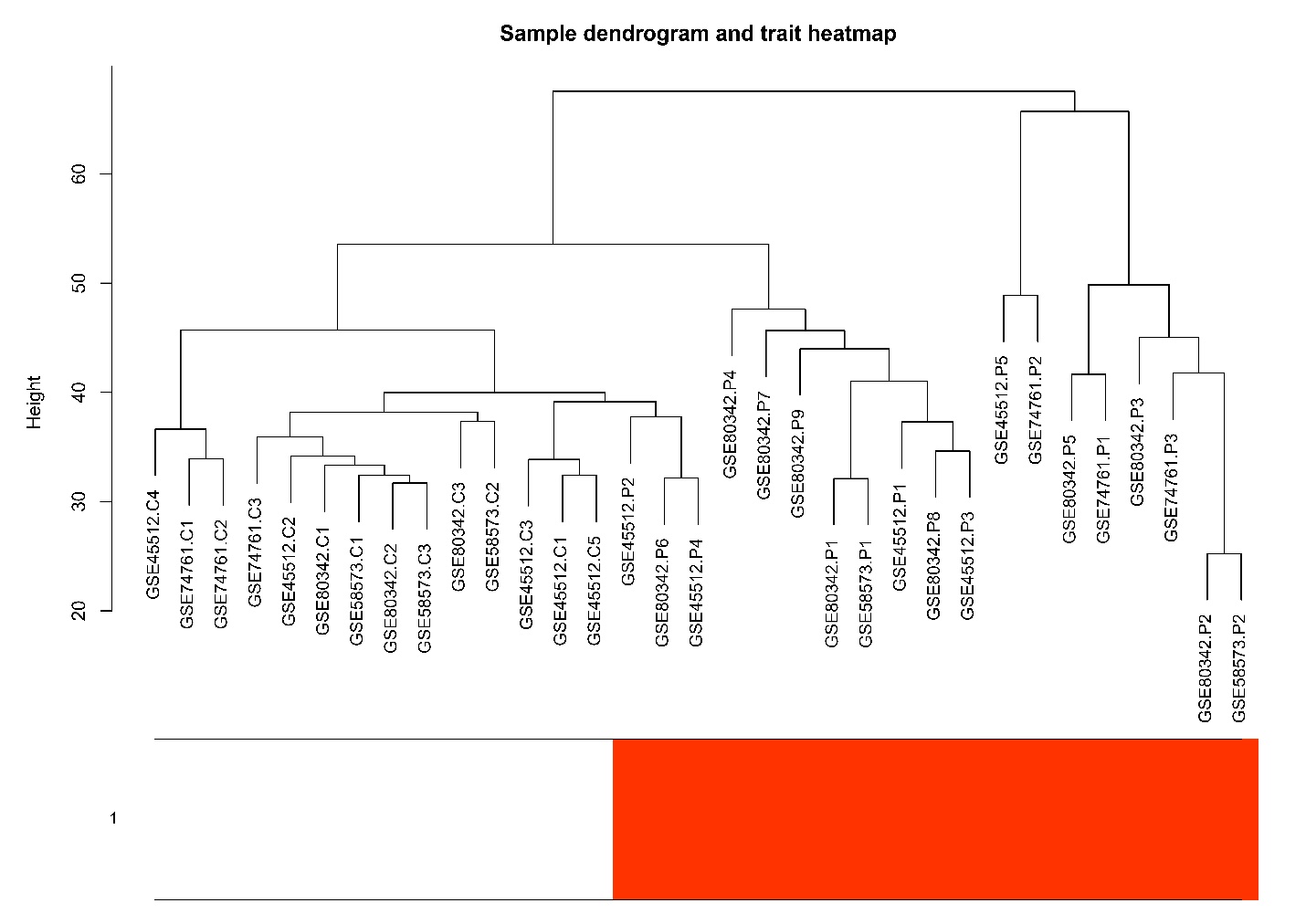


Control

Patient

**Fig S2:**


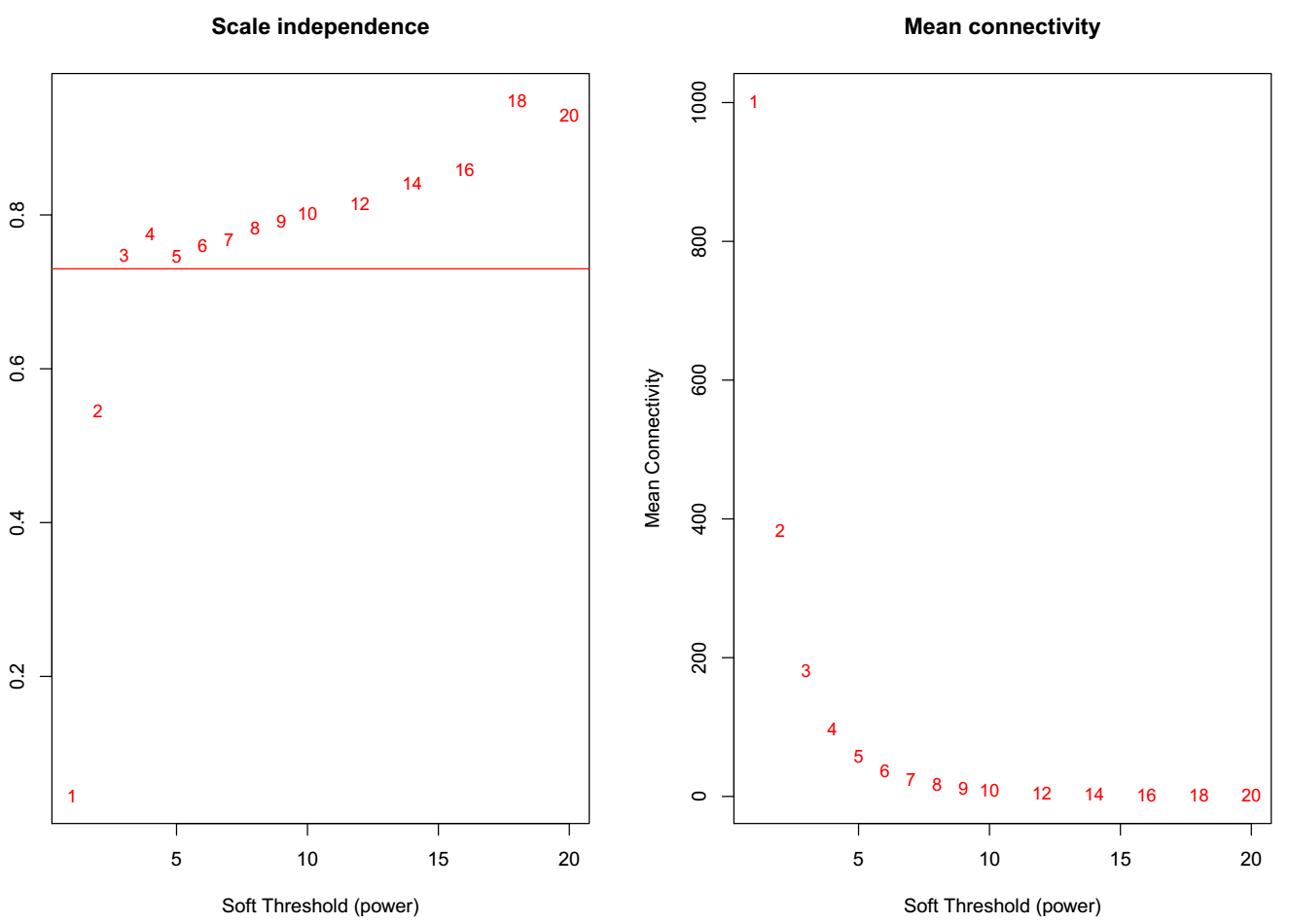


**Fig S3:**


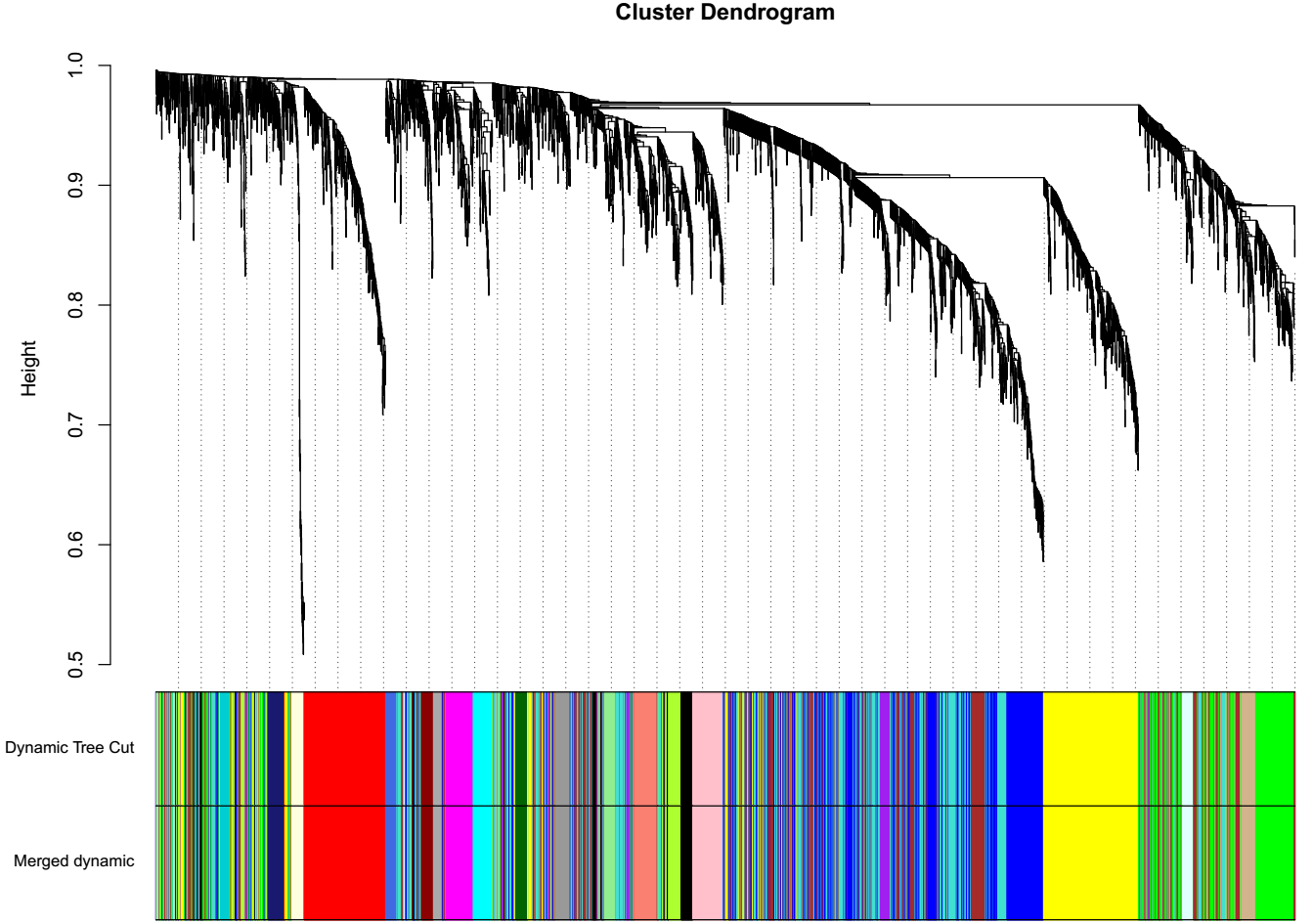

Supplement: Supplementary file 1 — Supplementary Material S1 [file SYB2-16-173-s001.docx]
